# Submarine mud volcanoes as a source of chromophoric dissolved organic matter to the deep waters of the Gulf of Cádiz

**DOI:** 10.1038/s41598-021-82632-3

**Published:** 2021-02-05

**Authors:** Valentina Amaral, Cristina Romera-Castillo, Jesús Forja

**Affiliations:** 1 Departamento de Química-Física, INMAR, Universidad de Cádiz, Puerto Real, España; 2 Ecología Funcional de Sistemas Acuáticos, Centro Universitario Regional Este, Universidad de La República, Rocha, Uruguay; 3 Instituto de Ciencias del Mar-CSIC, Barcelona, España

**Keywords:** Biogeochemistry, Ocean sciences

## Abstract

Seafloor structures related to the emission of different fluids, such as submarine mud volcanoes (MVs), have been recently reported to largely contribute with dissolved organic matter (DOM) into the oceans. Submarine MVs are common structures in the Gulf of Cádiz. However, little is known about the biogeochemical processes that occur in these peculiar environments, especially those involving DOM. Here, we report DOM characterization in the sediment pore water of three MVs of the Gulf of Cádiz. Estimated benthic fluxes of dissolved organic carbon (DOC) and chromophoric DOM (CDOM) were higher than in other marine sediments with an average of 0.11 ± 0.04 mmol m^−2^ d^−1^ for DOC and ranging between 0.11 and 2.86 m^−1^ L m^−2^ d^−1^, for CDOM. Protein-like components represented ~ 70% of the total fluorescent DOM (FDOM). We found that deep fluids migration from MVs (cold seeps) and anaerobic production via sulfate-reducing bacteria represent a source of DOC and FDOM to the overlying water column. Our results also indicate that fluorescent components can have many diverse sources not captured by common classifications. Overall, MVs act as a source of DOC, CDOM, and FDOM to the deep waters of the Gulf of Cádiz, providing energy to the microbial communities living there.

## Introduction

Dissolved organic matter (DOM) is one of the Earth’s major carbon reservoirs and the largest ocean pool of reduced carbon (662 Pg C)^1^. The fraction of DOM that absorbs light, referred to as chromophoric DOM (CDOM), and its fluorescent sub-fraction (FDOM) are ubiquitous constituents of the ocean DOM pool, affecting the optical properties of the water column^2,3^. The major sources of marine DOM are photosynthesis from the ocean surface and terrestrial inputs, however, marine sediments are also considered to be an important source of DOM to the overlying seawater (~ 350 Tg C y^−1^)^4^, comparable to that estimated for rivers (~ 250 Tg C y^−1^)^5^. Marine sediments are also a source of CDOM, but there is comparatively little information about the benthic flux of CDOM and the nature of these compounds^6^. Recent studies suggest that seafloor features related to large emissions of different fluids can act as sources of DOM and CDOM into the oceans^7–9^ therefore, having a significant influence on the chemistry of the oceans^10^.

Seafloor fluids can derive from microbiological and geological processes and they are classified in hydrothermal (40 °C up to 400 °C) and cold seeps (< 60 °C). Hydrothermal vents have been described as sinks of DOC, removing 1.4 ± 0.7 × 10^4^ tons C yr^−1^ from the deep-ocean^11,12^. However, they have been also found to act as a source of CDOM to the seawater^9^. Cold seeps comprise brines, methane and other hydrocarbons and are mainly controlled by tectonics^13^, but there is little information regarding the behavior of DOM from these fluids. Submarine cold seepage could result in a variety of seafloor structures such as mud volcanoes (MVs).

Submarine MVs are broadly distributed on Earth continental shelves, insular slopes and abyssal parts of inland seas (10^3^–10^5^)^14^. MVs are cone-shaped structures built up by mud breccia containing gases (mainly methane), saline water, mud, and, occasionally, oil rising from deep pressurized sources through controlled conduits^15,16^. They can be associated with the presence of chemosynthetic biological communities^17,18^. A recent study in the South China Sea suggests that MVs are an important source of DOC to the deep waters through benthic fluxes, and these could represent up to 24% of the total annual flux from the largest river that drains into this sea^19^. Moreover, Retelleti et al.^20^ also measured a high concentration of DOC and FDOM in pore water from MVs (1–6 mM and 0.01–0.06 RU, respectively). The authors found that fluid migrating from deep sediments through MVs could be an important source of altered FDOM to the overlying water column, with a total fluorescence of up to eight times higher than sediments without MVs. They suggest that these structures may play a significant role in providing FDOM to the ocean. However, as far as we know, no studies have estimated benthic fluxes of CDOM and FDOM from MVs yet, and their role in the deep ocean is poorly understood.

The Gulf of Cádiz is a tectonically active area where large fields of submarine MVs, associated with tectonic compression due to the convergence of the African and the Eurasian plates^21^, have been recently discovered^17,22,23^. Its geological features, the geochemistry and emission of fluids, and the benthic fauna associated with these fluids have been widely studied^16,17,24,25^. However, no studies have focused on the dynamic and diffusive benthic flux of DOM from these MVs.

**Figure 1:**
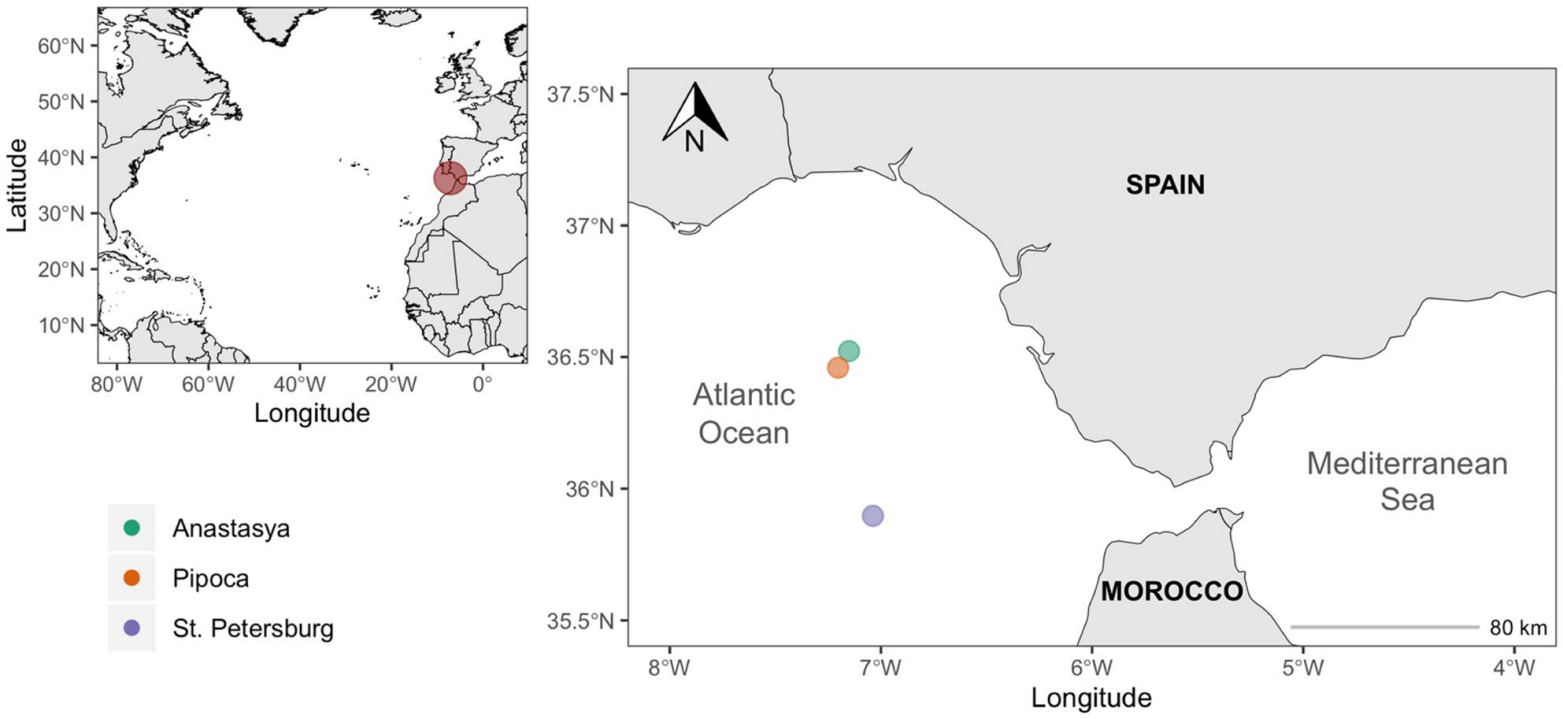
Map of the Gulf of Cádiz showing the location of the mud volcanoes.

This work aims to quantify and characterize DOM in the pore water of three MVs located in the Gulf of Cádiz (Fig. 1), and to determine the possible sources of the different fractions of DOM. To do so, we measured DOC concentration and DOM optical properties (absorbance and fluorescence). We also estimated diffusive benthic fluxes of DOC, CDOM, and FDOM across the sediment–water interface at these MVs to better understand their effect on the concentration and distribution of DOM into the deep water of the Gulf of Cádiz.

## Results

### Sediment pore water profiles of DOM and proxy of MVs fluids

Differences between cruises in pore water samples were considered to be due to spatial heterogeneity previously described in MVs^16^. During December 2016, it was not possible to sample St. Petersburg (PT) due to weather conditions. Overall, similar values between cruises were observed in Pipoca (PI) and Anastasya (AN) (*p* > 0.05). Only DOC concentration and BIX values were higher in AN during June (*p* < 0.05). Thus, for simplicity, only the pore water profiles of June will be presented, and those from December can be found in the Supplementary material (Fig. S1 and S2).

**Figure 2:**
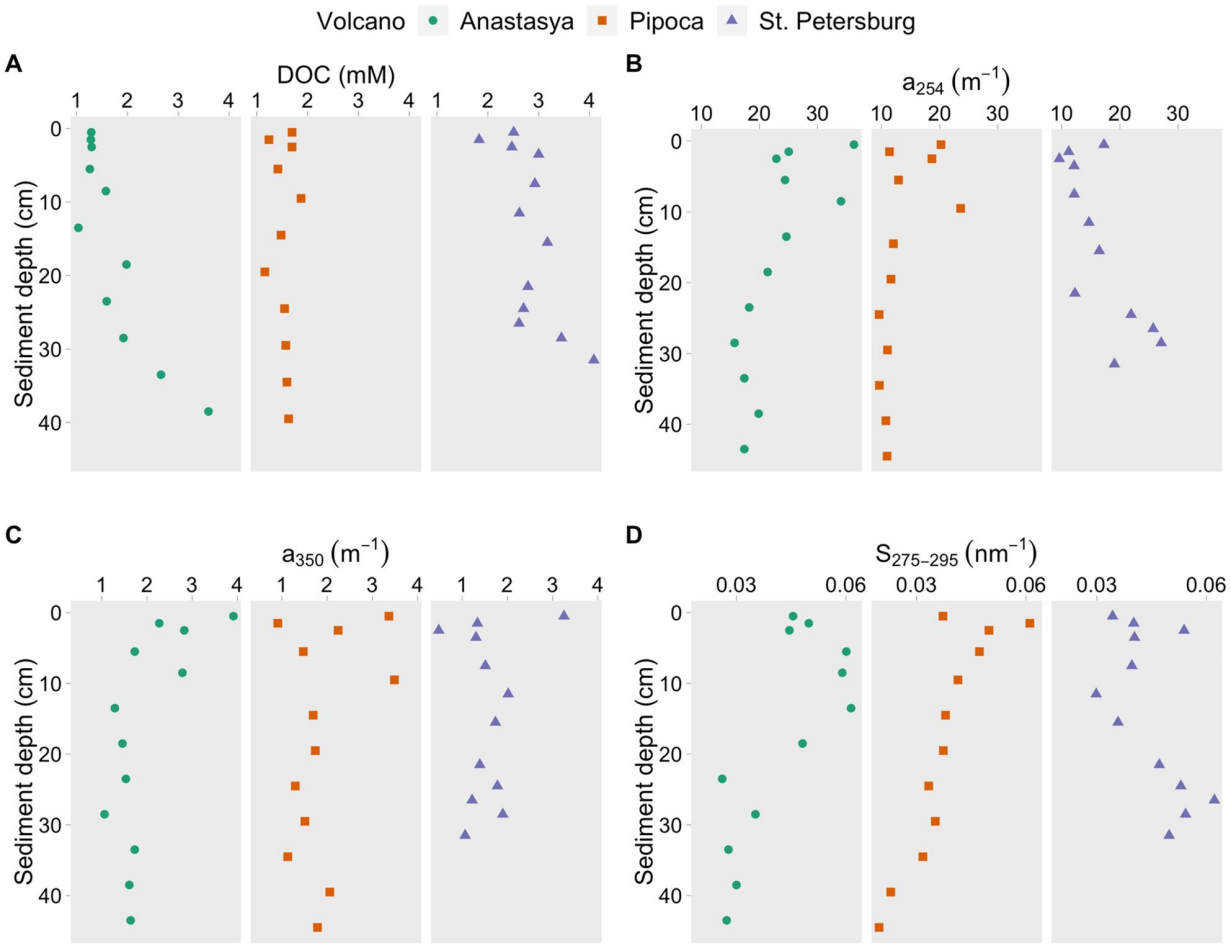
Pore water profiles of dissolved organic carbon (**A**: DOC), absorption coefficients at 254 nm (**B**) and 350 nm (**C**) and the spectral slope S_275-295_ (**D**) in the three mud volcanoes.

DOC concentration in pore water ranged from 0.63 to 4.08 mmol L^−1^, with higher values in PT (Table 1, *p* < 0.001) and increasing with depth in the three MVs (Fig. 2A). The absorption coefficient *a*_254_ ranged between 9.58 and 36.25 m^−1^, with higher values in AN (Fig. 2B,* p* < 0.001) while *a*_350_ showed similar variations between MVs (Fig. 2C, 0.48–3.91 m^−1^, *p* > 0.05). Although the spectral slope S_275-295_ showed similar values between MVs (Fig. 2D,* p* > 0.05), ranging from 0.020 to 0.062 nm^−1^, AN and PI exhibited an opposite trend when compared to PT. Thus, S_275-295_ decreased with depth in AN and PI while in PT, it increased. Average values of S_275-295_ showed that most of the DOM in the MVs was of relatively low molecular weight (Table 1).

**Table 1:** Pore water average ± standard deviation values of dissolved organic carbon (DOC, mmol L^−1^), absorption coefficient *a*_254_ and *a*_350_ (m^−1^), the spectral slope S_275-295_ (nm^−1^), fluorescent components (C1 to C4, RU), humification (HIX) and freshness (BIX) indexes during June and December 2016 in Anastasya and Pipoca and during June 2016 in St. Petersburg. Interval variations are shown in brackets.

	Anastasya	Pipoca	St. Petersburg
DOC	1.44 ± 0.67 (0.63–3.59)	1.50 ± 0.43 (0.74–2.43)	2.85 ± 0.56 (1.83–4.08)
*a*_254_	24.36 ± 5.02 (15.71–36.25)	14.17 ± 3.59 (9.59–23.59)	16.63 ± 5.79 (9.58–27.10)
*a*_350_	1.93 ± 0.63 (1.06–3.91)	1.67 ± 0.66 (0.91–3.49)	1.59 ± 0.67 (0.48–3.25)
S_275-295_	0.045 ± 0.012 (0.021–0.061)	0.038 ± 0.009 (0.020–0.061)	0.045 ± 0.010 (0.030–0.062)
C1	0.25 ± 0.17 (0.09–0.82)	0.18 ± 0.08 (0.09–0.40)	0.37 ± 0.016 (0.17–0.66)
C2	0.36 ± 0.24 (0.10–0.93)	0.14 ± 0.02 (0.10–0.18)	0.13 ± 0.06 (0.08–0.31)
C3	0.55 ± 0.31 (0.32–1.70)	0.21 ± 0.14 (0.09- 0.70)	0.29 ± 0.16 (0.10–0.58)
C4	1.58 ± 1.03 (0.51–4.07)	0.39 ± 0.25 (0.18–1.25)	0.62 ± 0.41 (0.23–1.36)
HIX	0.59 ± 0.12 (0.35–0.75)	0.74 ± 0.04 (0.61–0.80)	0.78 ± 0.07 (0.70–0.80)
BIX	1.19 ± 0.59 (0.75–3.42)	0.80 ± 0.07 (0.65–0.90)	0.80 ± 0.11 (0.57–0.98)

Parallel factor analysis (PARAFAC) of pore water samples from the three MV resulted in a four-fluorescent-components model (Fig. S3). The components were compared with previous studies in the OpenFluor database with a Tucker Congruence Coefficient (TCC) > 0.97 (Table S2)^26^. Component 1 (C1) and 2 (C2) were previously described as humic-like components of terrestrial and marine/microbial origin, respectively. Component 3 (C3) and component 4 (C4) were referred to as protein-like components, accounting for 68% ± 17% of the total FDOM. C4 resembles the amino acid tyrosine, and C3 is less common than other components. Amaral et al.^27^ found a similar component to our C3 in the water column of the Gulf of Cádiz (labeled as C5) and they suggested it could be a mixture of polycyclic aromatic hydrocarbon (PAH) and protein-like substances.

**Figure 3:**
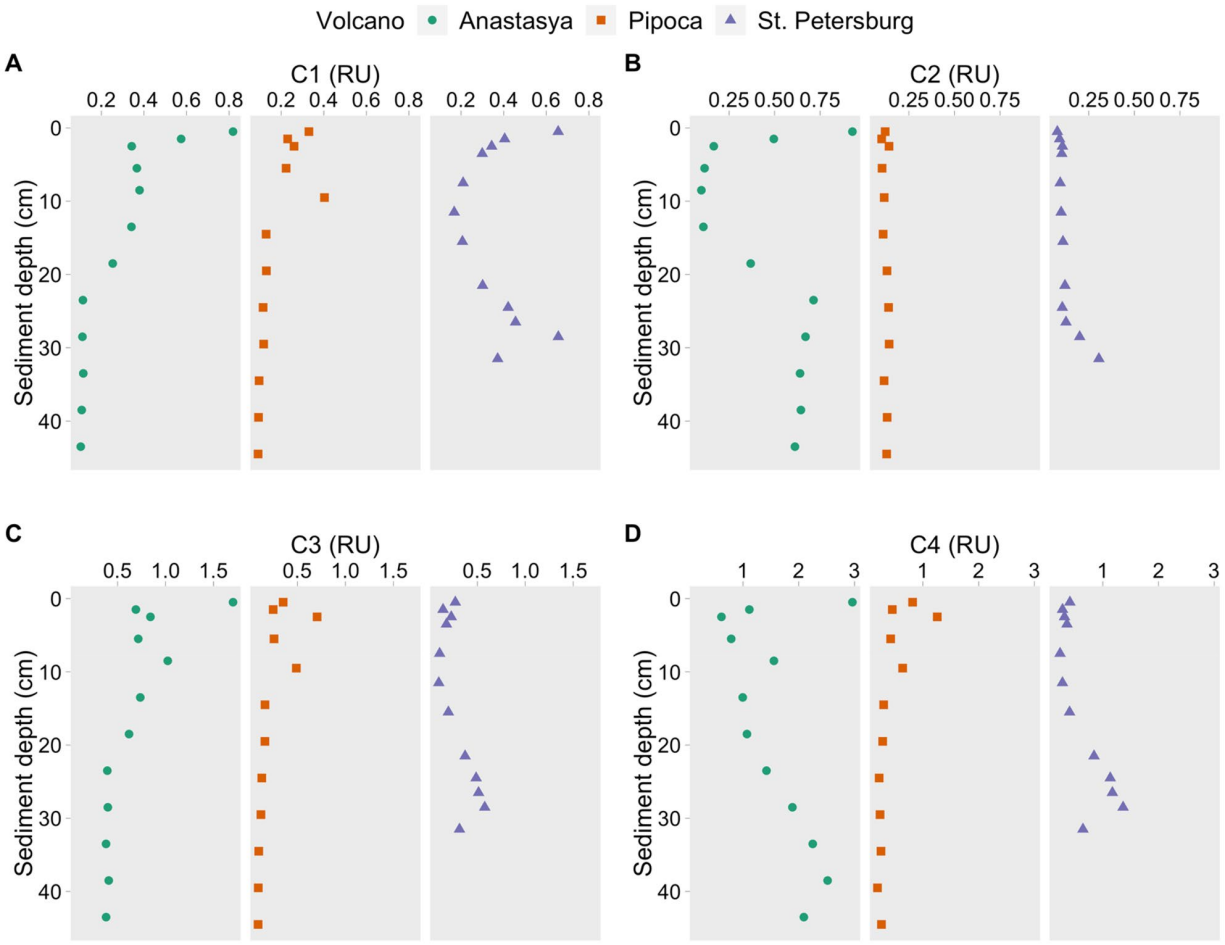
Pore water profiles of the PARAFAC fluorescent components (**A**: C1, **B**: C2, **C**: C3 and **D**: C4) in the three mud volcanoes.

Humic-like C1 showed similar values between MVs (Table 1, Fig. 3A,* p* > 0.05). The rest of the fluorescent components showed the highest intensities and variability in AN, with average values two and three-fold times higher than in the other MVs (*p* < 0.001). In PI and PT, C2 and C3 had similar constant vertical distribution with a slightly increase of C2 below 30 cm in PT (Fig. 3B,C). C4 also increased downward in PT while in PI, it remained relatively constant with depth (Fig. 3D). In AN, maximum values of C2, C3, and C4 were found in the first 0.5 cm, which sharply decreased until 2.5 cm, and below 13 cm C2 and C4 increased again downward while C3 remained relatively constant.

Only AN exhibited a linear relationship between total fluorescence (C1 + C2 + C3 + C4) and DOC, with a y-intercept of 0.49 mmol L^−1^ (*p* < 0.01, n = 22), which is the colorless fraction of DOM. Since the average DOC concentration in AN was 1.44 mmol L^−1^, we estimated that 66% of the DOC pool in the pore water of this mud volcano is composed of fluorescent material.

The humification and freshness indexes (HIX and BIX) showed an opposite pattern (Fig. S6) with the lowest HIX values and highest BIX values in AN (Table 1, *p* < 0.001). In PI and PT, BIX values were lower than 1, while in AN, the average value was > 1.

Linear relationships between C1 with C2 and C4 revealed differences in their dynamics depending on the mud volcano (Table S3). C1 was negatively related with C2 and C4 in AN (R^2^ = 0.71 and 0.53, *p* < 0.01), but positively in PI (R^2^ = 0.71 and 0.82, *p* < 0.01) and PT (R^2^ = 0.60 and 0.62, *p* < 0.01), while it was positively related with C3 in the three MV (R^2^ = 0.71 and 0.53, *p* < 0.01). The other fluorescent components only exhibited significant linear relationships in PI and PT. Thus, the protein-like C3 and C4 showed a strong positive relationship between them and to a lesser exert with C2. *a*_254_ showed a positive relationship with all fluorescent components in PI and PT, but only with C1 and C3 in AN (R^2^ > 0.21, *p* < 0.01). No significant relationships were observed with *a*_350_ (*p* > 0.01).

**Figure 4:**
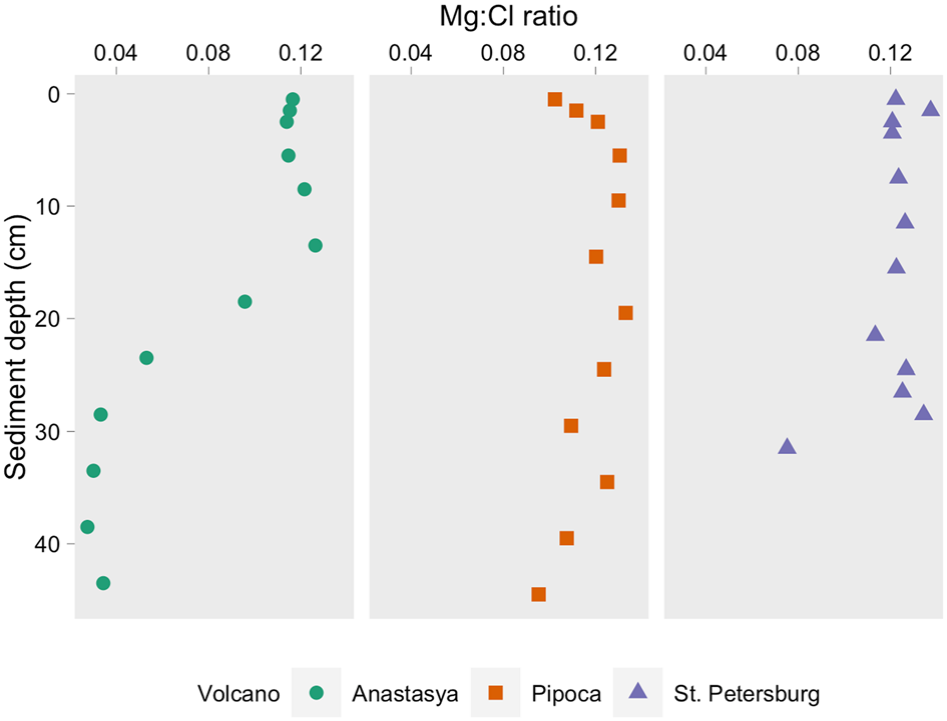
Pore water profiles of the Mg:Cl ratio in the three mud volcanoes.

Chloride (Cl) and magnesium (Mg) were measured in pore water samples and are described elsewhere (Jiménez-López et al. submitted)*.* Here, the Mg:Cl ratio was considered as a mixing indicator of seawater and MVs fluids (Fig. 4). The smaller the ratio, the higher it will be the fraction of MVs fluid in the pore water samples^22^. Similar values were observed in the uppermost samples from the three MVs (0.5 cm, 0.11 ± 0.01), which mainly correspond to the hemipelagic sediments. The Mg:Cl ratio decreased with depth in AN, reaching values of 0.03, even smaller than those observed in other active MVs^22^. Conversely, in PI and PT, the ratio remains relatively constant, except for the last depth in PT where it decreases to 0.07 (31 cm). Similar trends and values were observed between sampling periods (*p* > 0.05).

### Seawater profiles of DOM in the water column above the MVs

The concentration of DOC, *a*_254,_ and *a*_350_ were one and two orders of magnitude lower than those observed in pore water, ranging between 0.04 to 0.14 mmol L^−1^, 0.88 to 2.03 m^−1^, and 0.01 to 0.4 m^−1^, respectively (Fig. S4, *p* < 0.05). The spectral index S_275-295_ ranged between 0.018 and 0.045 nm^−1^, with an average value of 0.028 ± 0.007 nm^−1^, lower than those observed in pore water samples (0.043 ± 0.01 nm^−1^).

For the water column samples, the PARAFAC model validated three largely described components in aquatic systems, a humic-like and two protein-like components. The TCC between the fluorescent components in the water column and pore water was < 0.95, implying that the components compared cannot be considered equal^28^. Therefore, to compare the FDOM between both datasets, we applied the pick picking technique in the EEMs from the water column^29^ using the same Ex/Em wavelengths of the PARAFAC components identified in the pore water samples. Fluorescence intensity was tenfold higher in pore water than in the water column. C1 followed the same trends in the three sites (*p* > 0.05), increasing with depth (Fig. S5) with a strong relationship with temperature and salinity (R^2^ = 0.87, *p* < 0.01). In the water column, C2 and C3 were higher in AN than the other sites (*p* < 0.05), increasing sharply with depth, associated with the thermocline and the peak of Chl *a* ^27^, while in PI and PT, they remained relatively constants. C4 exhibited an irregular profile in the three sites (*p* > 0.05), and an increase of C2, C3, and C4 was observed in the summit of AN and PT (Fig. S5).

### Estimated diffusive benthic fluxes of DOC, CDOM, and FDOM

All variables showed positive benthic flux toward the water column (Table 2). The estimated benthic fluxes of DOC varied between 0.07–0.18 mmol m^−2^ d^−1^, being highest in PT. The estimated benthic fluxes of CDOM, as *a*_254_ and *a*_350_, ranged from 1.15 to 2.86 and from 0.11 to 0.31 (m^−1^ L m^−2^ d^−1^), respectively, with higher values in AN. The humic-like C1 showed similar fluxes between MVs ranging from 0.02 to 0.05 (RU L m^−2^ d^−1^), while C2 ranged between 0.008 and 0.070 (RU L m^−2^ d^−1^) with the highest value in AN. Protein-like C4 showed the highest fluxes among the fluorescent components ranging from 0.02 to 0.240 RU L m^−2^ d^−1^, with the highest value also in AN.

**Table 2:** Estimated diffusive benthic fluxes of DOC, CDOM, and FDOM in the three MVs.

Variable	Studies	Range	WD (m)
DOC (mmol m^−2^ d^−1^)	Anastasya	0.07–0.1	457
	Pipoca	0.08–0.13	503
	St. Petersburg	0.18	860
	MVs from China Sea^a^	0.03–0.52	367–668
	Weddell Sea^b^	0.1–0.54	316–494
	European margins^c^	0.05–0.16	180–4800
	N Atlantic Ocean^d^	0.08	300–1000
	Arabian Sea^e^	0.060–0.22	> 3000
	NE Atlantic Ocean^e^	0.05–0.12	> 4000
	Estuarine^f^	0.96–1.10	12–15
*a*_254_ (m^−1^ L)(m^−2^ d^−1^)	Anastasya	2.12–2.86	
	Pipoca	1.50–1.54	
	St. Petersburg	1.15	
*a*_350_ (m^−1^ L)(m^−2^ d^−1^)	Anastasya	0.15–0.31	
	Pipoca	0.11–0.26	
	St. Petersburg	0.22	
	Arctic Ocean^g^	− 0.03–0.18	100–2240
C1 (RU L)(m^−2^ d^−1^)	Anastasya	0.03–0.05	
	Pipoca	0.02	
	St. Petersburg	0.04	
	Arctic Ocean^g^	0–0.07	100–2240
	Estuarine^f^	0.08–3.41	12–15
C2 (RU L)(m^−2^ d^−1^)	Anastasya	0.02–0.07	
	Pipoca	0.01	
	St. Petersburg	0.008	
	Arctic Ocean^g^	0.02–0.06	100–2240
	Estuarine^f^	0.08–3.97	12–15
C3 (RU L)(m^−2^ d^−1^)	Anastasya	0.02–0.13	
	Pipoca	0.003	
	St. Petersburg	0.01	
	Estuarine^f^	0.19–6.09	12–15
C4 (RU L)(m^−2^ d^−1^)	Anastasya	0.05–0.24	
	Pipoca	0.05–0.07	
	St. Petersburg	0.03	
	Arctic Ocean^g^	− 0.04–0.03	100–2240
	Estuarine^f^	0.19–6.09	12–15

Fluxes from other regions are also displayed (WD: water column depth).

^a^Hung et al.^19^; ^b^Hulth et al.^58^; ^c^Otto and Balzer^59^; ^d^Alperin et al.^60^; ^e^Lhajnar et al.^61^; ^f^Burdige et al.^35^*; ^g^Chen et al.^36^. Positive fluxes are out of the MVs. *1 RU = 64 μg QS.

## Discussion

The fluorescent components found in the pore water model have been described for several aquatic environments (Table S2). Our results support the hypothesis that similar components may have different sources^6,30^. In the water column above the MVs, C1 and C2 showed the usual positive relationship between humic-like components from similar sources (R^2^ = 0.85, *p* < 0.001, n = 37), and mixing processes explained their distribution, in agreement with previous studies in this zone^27^. The same occurs with the protein-like components, where C3 and C4 were positively related (R^2^ = 0.60, *p* < 0.001, n = 37), and probably associated with biological activity^27^. Conversely, different behaviors were observed in pore water samples from the MVs. In AN, humic-like C1 and C2 were negatively related (R^2^ = 0.71, *p* < 0.001, n = 58), and no relationship was observed between protein-like C3 and C4, indicating differences in their sources and sinks. On the other hand, all fluorescent components in PI and PT, showed a positive relationship among them (R^2^ > 0.50, *p* < 0.001, Table S3). Therefore, the processes that produce and remove FDOM also differ between MVs.

One possible source could be the production of thermogenic DOM together with the production of methane. More than 2.4% of DOM compounds in the ocean are thermogenic^7^, and a previous work suggests that the FDOM coming from the deep sediments of MVs fluids was mainly thermogenic^20^. According to the authors, the lower DOC-normalized fluorescence in the MVs, compared to those found in a reference site (without MVs), may be due to the thermal quenching of the fluorophores. Based on stable carbon isotope composition of dissolved methane in the column water above the MVs studied here, Sierra et al.^31^ found that methane emissions in AN and PT are of thermogenic origin, while in PI, it seems to be of biogeochemical origin. Unfortunately, we did not measure DOM composition of other pore water sediments in the zone to use as a reference site, and similar values of DOC-normalized fluorescence were observed between AN, PT and PI. Thus, a thermogenic origin of FDOM in this work cannot be confirmed.

**Figure 5:**
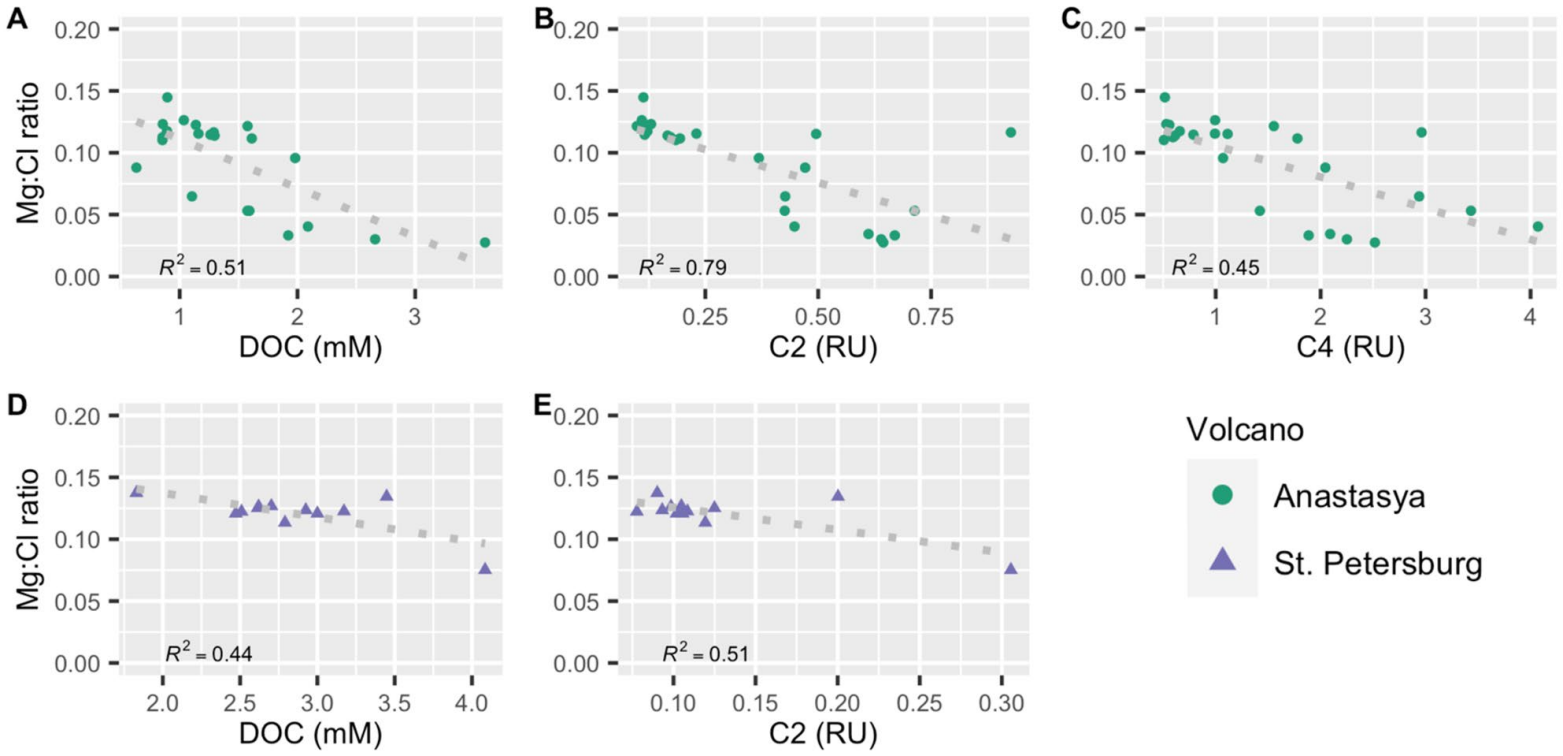
Linear relationship between the Mg:Cl ratio with dissolved organic carbon (DOC) and fluorescent components in Anastasya (**A**–**C**) and St. Petersburg (**D**,**E**). Only the relationships with *p* < 0.01 are shown.

However, the differences in the DOM sources between MVs could be related to differences in their activity stage, hence, with the intensity of the fluids moving upward from the deeper sediments. MVs alternate between massive mudflow extrusion and dormant/sleeping periods^17^. According to Mazzini et al.^18^, most of the MVs are currently in the latter, generally characterized by gas and water seepage of variable intensity. The Mg:Cl ratio indicated a higher fraction of MVs fluid in AN than in the other MVs (Fig. 4) and Sierra et al.^31^ found an increase of methane with depth in the water column above AN (100–125 nmol L^−1^) and PT (10–15 nmol L^−1^), whereas in PI this trend was not observed. This is in agreement with a previous work analyzing benthic fauna associated with seepage of MVs fluids, suggesting locally elevated gas fluxes that reach the seafloor surface, and higher activity in AN than PI^17^. Additionally, habitats with structures created by the escaping gas were also found in PT^32^, and elevated methane fluxes have also been found in relatively active MVs in the Gulf of Cádiz^16,33^. The level of extrusion activity between the MVs studied here is AN > PT > PI and it could be the reason for the existence of different sources of DOM in the MVs. For example, in AN, DOC, C2 and C4 exhibited a negative relationship with the Mg:Cl ratio (R^2^ = 0.51, 0.79, 0.45, respectively, *p* < 0.001, n = 23, Fig. 5), indicating an increase with the increasing mud volcano fluids. In PT, DOC and C2 also showed an inverse relationship with this ratio, weaker than in AN, but significant (R^2^ = 0.44 and 0.51, respectively, *p* < 0.01, n = 12, Fig. 5), while no relationships were observed in PI (*p* > 0.01). Our results indicate that cold seeps from MVs could be a source of DOC and FDOM to shallower sediments and, eventually, to the overlying water column.

**Figure 6:**
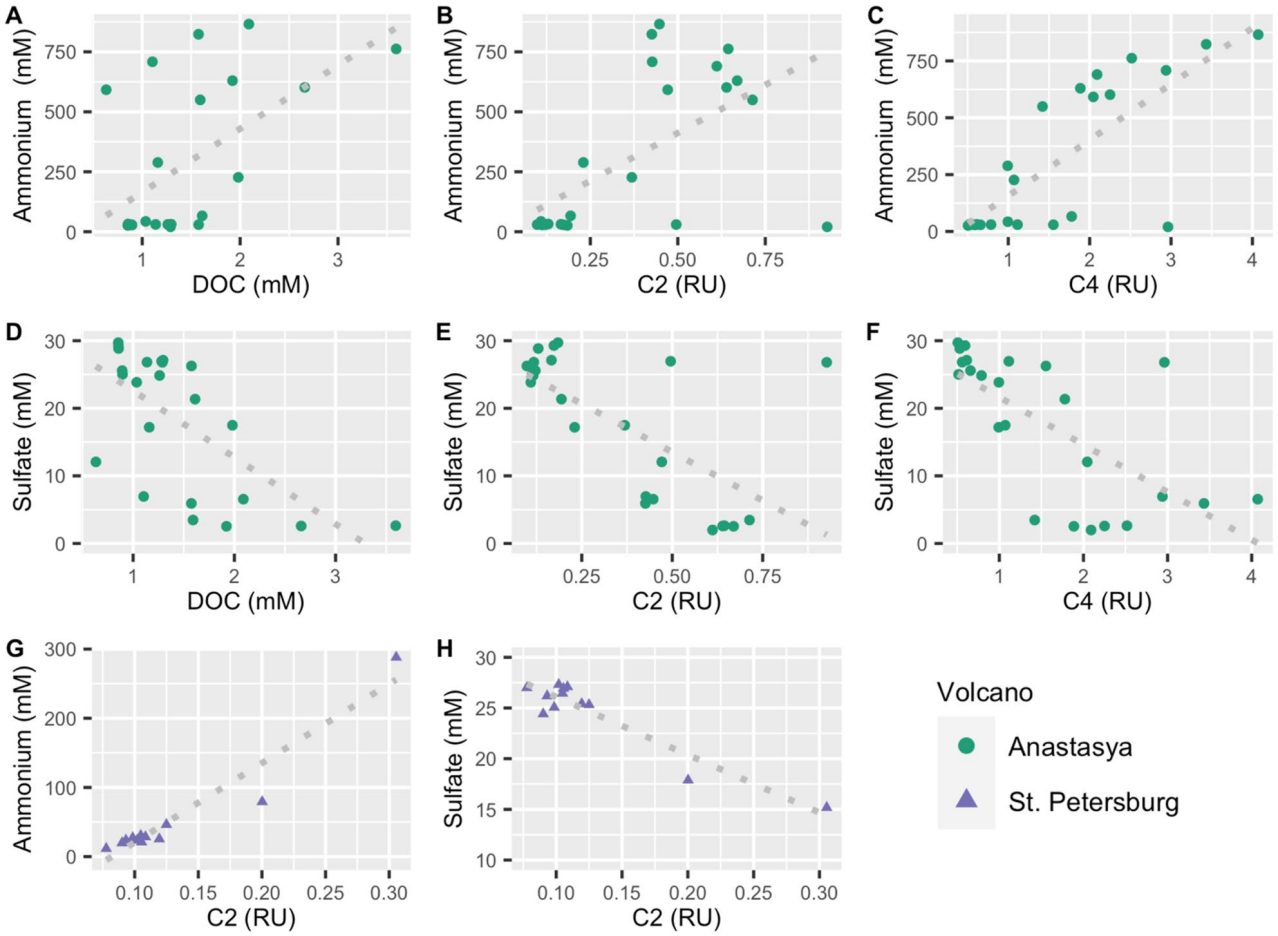
Linear relationship between ammonium and sulfate with dissolved organic carbon (DOC) and fluorescent components C2 and C4 in Anastasya (**A**–**F**) and St. Petersburg (**G**,**H**). Only the relationships with *p* < 0.01 are shown.

We found a net production of DOC, CDOM, and FDOM to the overlying water column in the three MVs (Table 3). However, as previously discussed for PI, and given the R^2^ of the relationship between DOM and the Mg:Cl ratio found in AN and PT, there might be other factors and sources implicated in the final distribution of DOM. In sediments, there is generally a net production of DOM as a result of organic matter degradation processes^4^. Sulfate is one of the main electron acceptors available for the oxidation of organic matter in marine sediments; hence sulfate reduction is an important diagenetic process below the oxic zone^34^. Several studies in marine sediments found that FDOM could be produced via anaerobic degradation of particulate organic matter through sulfate reduction or oxidation of methane^35–37^. Luek et al.^30^ found that the production of FDOM was directly related to sulfate-reducing bacteria in incubations of coastal sediments. The decomposition of organic matter by sulfate reduction consumes sulfate and releases ammonium^34^. Both sulfate and ammonium were measured in pore water samples from the three MVs and are described elsewhere (Jiménez-López et al. submitted). Accordingly, in AN, the increase of DOC concentration, C2 and C4 was accompanied by the increase in ammonium (Fig. 6A–C, R^2^ = 0.28, 0.75 and 0.79, respectively, *p* < 0.01, n = 23) concurrent with the decrease of sulfate (Fig. 6D–F, R^2^ = 0.39, 0.89 and 0.59, respectively, *p* < 0.01, n = 23). Moreover, in AN, these fluorescent components were related to a decrease in DOM humification and with an increase of freshly released DOM (Table S3). In PT, only C2 exhibited a strong relationship with ammonium and sulfate, with the same trends as in AN (Fig. 6G,H, R^2^ = 0.91 and 0.87, respectively, *p* < 0.01, n = 12), whereas no relationship was observed in PI (*p* > 0.01). These results suggest that DOC concentration, C2 and C4 could also be related to the activity of sulfate-reducing bacteria in MVs sediments.

**Table 3:** Production of dissolved organic carbon (DOC, mmol L^−1^), chromophoric DOM (as *a*_254_ and *a*_350,_ m^−1^) and fluorescent components (C1 to C4, RU) in the pore water sediments of Anastasya (AN), Pipoca (PI) and St. Petersburg (PT) during June and December 2016.

MV Cruise	DOC	*a*_254_	*a*_350_	C1	C2	C3	C4
AN June	3.5	16.0	1.5	0.1	0.6	0.3	2.0
AN Dec	2.0	30.9	1.8	0.1	0.4	0.4	4.0
PI June	1.6	9.6	1.6	0.1	0.1	0.1	0.2
PI Dec	2.1	12.2	1.1	0.1	0.1	0.1	0.2
PT June	4.0	17.8	0.9	0.4	0.3	0.3	0.6

The net increase is indicated (= value bottom depth pore water − value bottom water).

Additionally, AN is characterized by the presence of large microbial mats in its summit, associated with cold seep and extremophiles conditions in its anoxic sediments, while chemosynthetic-based communities have also been found in PT^17,38^. The biological release of DOC has been previously reported in seep environment characterized by the presence of microbial mats in the Hydrate Ridge^39^. The deep DOC in fluid seepage may stimulate the microbial community to release autochthonous organic matter and enhance heterotrophy in seep environments^39^. Based on BIX values, Retelleti et al.^20^ suggested that MVs have more abundance of DOC from biological sources than sediments without MVs, which has been attributed to the direct release of DOC from microorganisms. This could explain the highest BIX values observed in AN (> 1 up to 3), which corresponds to the presence of DOM recently released^40^. Anaerobic production of DOM via sulfate-reducing bacteria might also be a source of DOM in AN and, to a lesser extent, in PT, providing energy to the microbial communities living there. These processes were also suggested as one of the responsible of FDOM composition in other MVs^20^.

On the other hand, deep cold seep fluids and biological activity appear to be a minor source of DOM in PI, probably linked to the lower seepage activity of this mud volcano, as previously mentioned. Although PI is located in the same MVs field than AN (Table S1), it is strongly influenced by bottom currents from Mediterranean waters^32^ and organisms not associated with fluid emissions, but typical in these waters, such as dense crinoid beds, had been observed in its summits^17^. We hypothesized that the net increase of DOM observed in PI (Table 3) could be due to abiotic processes such as condensation, fragmentation, or physical dissolution of POM to DOM^3,41^. For example, abiotic condensation of FDOM could be responsible of refractory organic matter^3^. This could explain the negative relationship between FDOM components with BIX observed in PI, but not observed in the other MVs (Table S3), as well as the relatively less variable DOM profiles in pore water (Table 1, Figs. 2 and 3).

Our results agree with previous works that suggest that similar fluorescent components may have different sources^6,30^. Humic-like C2 was first proposed as a component of marine origin^29^, but further studies indicate that this component is observed in almost all aquatic environments (Table S2), and its signal was then attributed to microbial humic-like fluorescence^3^. In the water column of the Gulf of Cádiz, C2 was described from both autochthonous and allochthonous sources^27^, while in this work it was related to cold seeps from MVs and bacterial sulfate-reduction. Several works indicate that ‘humic-like’ DOM is also produced in the absence of terrestrial sources or humification processes^3,30,42^. Our results support the hypothesis that spectral regions originally defined as “humic-like” can have many diverse sources not considered by the traditional classification^29^. On the other hand, the distribution of C1 might be explained by deposition from the water column and/or solubilization, given the similar distribution observed between MVs (*p* > 0.05), while the net increase observed might be due to abiotic processes. Another possibility could be C1 production by solubilization of the terrestrial organic matter that has settled.

Finally, C3 was described as a mixture of PAH and protein-like components related to anthropogenic pollution in the water column of the Gulf of Cádiz^27^. Although MVs could also be a source of PAH^18^, a unique source cannot be distinguished using PARAFAC. Spectral fluorescence overlapping between fluorophores with low excitation wavelength such as PAH^43^ and signals from proteins^44^ might result in a mixture of fluorophores. Moreover, Sharma et al.^45^ found a similar component to our C3 in soil organic matter and described it as a mixture of terrestrial humic-like and tryptophan fluorescence (peak-T)^29^ with almost no biological production, degradation or absorption. Accordingly, C3 was positively related to the terrestrial C1 in the three MVs (Table S3) and also with the peak-T (R^2^ = 0.63, *p* < 0.01, n = 60) suggesting that C3 could be a mixture of fluorophores with different sources.

Continental margin sediments may be responsible for a significant fraction of DOM delivered to the marine environment^6,35,46^. The benthic fluxes of DOC were in the range of those estimated in other MVs and most continental slopes, and lower than those from estuarine sediments (Table 2). The highest estimated benthic flux of DOC was observed in PT, which could be due to the higher DOC concentrations observed here (Table 1). We can conclude that cold seep from MVs acts as a source of DOC to the deep waters, probably minimal compared to other oceanic sources (e.g. 250 × 10^6^ tons y^−1^ from rivers).

Although no information of diffusive benthic flux of CDOM and FDOM from MVs was found, our data was compared with those from marine sediments and hydrothermal vents. Benthic flux of CDOM (as *a*_254_ and *a*_350_) showed higher values than those reported for marine sediments, whereas the fluxes from the humic-like components were in the same range (Table 2). Among the fluorescent components, protein-like estimated benthic fluxes from AN were one order of magnitude higher than those in PI and PT and also in marine sediments. Our results suggest that MVs could act as a source of CDOM and FDOM to the deep waters of the Gulf of Cádiz. Noteworthy, benthic fluxes from a reference site without mud volcanoes in this zone will improve our findings.

## Conclusions

Cold seeps from MVs and biological activity via anaerobic sulfate-reducing bacteria represent a source of DOC, CDOM, and FDOM to the deep waters of the Gulf of Cádiz, providing energy and resources to the deep heterotrophic microbial communities. However, further studies are needed to better understand the role of mud volcanoes in DOM cycling in the deep water of the Gulf of Cádiz (e.g. stable isotopes, FT-ICR-MS, amino acid analysis).

## Methods

### Site description

The Gulf of Cádiz is located between the SW continental margin of the Iberian Peninsula and the NW margin of Africa (Fig. 1). It is strongly influenced by the exchange of Atlantic and Mediterranean water masses through the Strait of Gibraltar and it is characterized by the presence of several areas of hydrocarbon-rich fluid venting structures (e.g., MVs, pockmarks, carbonate chimneys and crusts, mud mounds and diapiric ridges)^17,22,47,48^.

The study was carried out in three MVs: Anastasya and Pipoca, situated in the Guadalquivir Diapiric Ridge Field, and St. Petersburg, in the deeper Tasyo field, located between 457 and 860 m depth (Table S1). Recent work found that bottom waters at these MVs exhibit high methane concentrations, especially in AN^31^. Based on cold seep fauna at the summit of these MVs and the characteristic of the mud breccia sediments (e.g., gas bubbles, smell of H_2_S), AN seems to be the more active one among the three MVs studied here^17^.

### Sampling and analytical methods

Two cruises were carried out onboard R/V Angeles Alvariño and Ramón Margalef during June and December 2016. Seawater samples from the water column were collected from 5 m to the summit of each MV (n = 44), and pore water samples were collected from gravity cores inside each MV. The 44 seawater samples were taken using Niskin bottles (10 L) mounted on a rosette-sampler coupled to a Seabird CTD 911 + to measure temperature and salinity. Details regarding the measurements of physicochemical variables are described elsewhere^27^. For measuring DOC concentration, absorbance and fluorescence, seawater was filtered using precombusted filters (Whatman GF/F, nominal pore size 0.7 µm, 450 °C, 4 h). Both optical analyses were performed on board with the same settings described below. Samples for DOC concentration were frozen in acid-clean HDPE bottles until analysis.

Two 1 m long gravity cores of 9.5 cm inner diameter were collected from inside each mud volcano. Subsamples from each core were taken onboard. Sections of 1 cm thickness were carefully sliced at different depths (~ 0.5 to 45 cm below the seafloor) and stored frozen until analysis. Sediments and pore water samples were treated in an inert atmosphere using N_2_ to minimize exposure to oxygen. Pore water samples were collected by centrifugation (~ 100 g of sediment, 30 min, 10.000 G and 10 °C, SIGMA-18KS, n = 60) and then filtered (Millipore HPF 0.45 µm). Combusted GF/F filters significantly increase their retention capacity due to compaction of the borosilicate microfibers^49^, hence no significant differences associated with using different filters for pore water and seawater samples are expected in this work. Even though centrifugation could have an effect on DOM composition, it would be minimal compared to other techniques when analyzing FDOM^50,51^. It does not require pretreatment and it is currently the most widely used method for collecting pore water from sediments for DOC and FDOM analyses (see references Table 2). Magnesium (Mg), chloride (Cl) and sulphate concentrations were measured in pore water samples by ionic chromatograph (Metrohm 881/882, Compact IC pro, plus, CV = 1.78 ± 0.26%), and ammonium was measured using a segmented flow auto-analyser (Skalar, San Plus) (Jiménez-López et al. submitted). For DOC analysis, pore water was diluted in MilliQ and stored frozen until analysis using a Multi N/C 3100 Analytik Jena analyzer (instrument variability = 43 ± 1.8 mM, Hansell CRM Program). UV–visible absorption spectra were immediately scanned from 250 to 800 nm using a spectrometer (JASCO-V750) using a 1 cm path length quartz cuvette. The estimated detection limit of the spectrophotometer for quantifying CDOM absorption is 0.0015 absorbance units or 0.03 m^1^ (1 cm cuvette), and the noise is 0.00004 absorbance units (specifications JASCO-750). A blank was measured every six samples to detect and correct instrument drift. We calculated the absorption coefficient at 254 and 350 nm and the spectral slope S_275-295_^52,53^. Excitation-emission matrixes (EEMs) at 250–450/300–560 nm were immediately obtained using a spectrofluorometer with an accessory for temperature control (JASCO FP-8300, EHC-813). Prior to PARAFAC modeling, EEMs from seawater and pore water samples were standardized (e.g. blank subtraction, instrument correction factor, Raman and Rayleigh scatter band were trimmed, and inner filter effect was corrected), and normalized to Raman Unit (RU) using the drEEM 0.2 toolbox for Matlab^54^. Fluorescent components were validated using split-half validation and random-initialization analysis^54^. We also calculated the humification (HIX) and freshness index (BIX) following Huguet et al.^40^. Further details for the calculation of spectral indexes, EEMs standardization, and PARAFAC analysis are described in a previous work^27^.

Assuming diffusive transport across the sediment–water interface, we estimated the benthic fluxes of DOC, CDOM (as *a*_254_ and *a*_350_), and FDOM components from the MVs using Fick’s first law of diffusion following Burdige et al.^35^ and Hung et al. ^19^.

$$J = \varphi Ds dC/dz$$

where φ is porosity, Ds is the bulk sediment diffusion coefficient corrected for sediment tortuosity (θ^2^) according to D_0_/θ^2^, D_0_ is the free solution coefficient (1.585 × 10^–6^ cm^2^ s^−1^)^55^ and θ^2^ was estimated using the modified Weissberg relationship (Boudreau^56^, θ^2^ = [1 − 2 Ln ($\varphi$)]). Finally, dC/dz was calculated as the difference between the bottom water and pore water concentration at 0.5 cm. It should be noted that bottom water collected at ~ 5 m above MVs was used, thus benthic flux estimation should be treated with a degree of caution, since, in general, water overlying the sediments (~ 30 cm) should be used^36^. To apply the same method, for FDOM we used the values obtained by pick picking techniques of the four PARAFAC components in both bottom and pore water samples.

### Statistical analysis

Two ways ANOVA and the post hoc Tukey test were performed to assess differences in DOM (factor 1: MVs and factor 2: cruises). A significance level of 0.05 was selected. Linear regression models were used to determine the relationship between DOM variables and were considered statistically significant when *p* < 0.01. All analyses were performed with R 1.3.1073 software^57^.

## Supplementary Information

Supplementary Information

## Data Availability

The datasets generated during the current study are available from the corresponding author on reasonable request.
