# Supplementary material for: Submarine mud volcanoes as a source of chromophoric dissolved organic matter to the deep waters of the Gulf of Cádiz
Source: Sci Rep. 2021 Feb 5;11:3200. doi: 10.1038/s41598-021-82632-3 (PMC7865021; doi:10.1038/s41598-021-82632-3)
Supplement: Supplementary file 1 — Supplementary Information [file 41598_2021_82632_MOESM1_ESM.pdf]

Supplementary Material

Submarine mud volcanoes as a source of chromophoric dissolved organic matter to the deep waters of the Gulf of Cádiz

Amaral, V.[1,2*], Romera-Castillo, C.[3], Forja, J.[1]

[1]Departamento de Química-Física, INMAR, Universidad de Cádiz, Puerto Real, España.
[2]Ecología Funcional de Sistemas Acuáticos, Centro Universitario Regional Este, Universidad de la República, Rocha, Uruguay.
[3]Instituto de Ciencias del Mar-CSIC, Barcelona, España.

***Corresponding author:** vamaral@cure.edu.uy

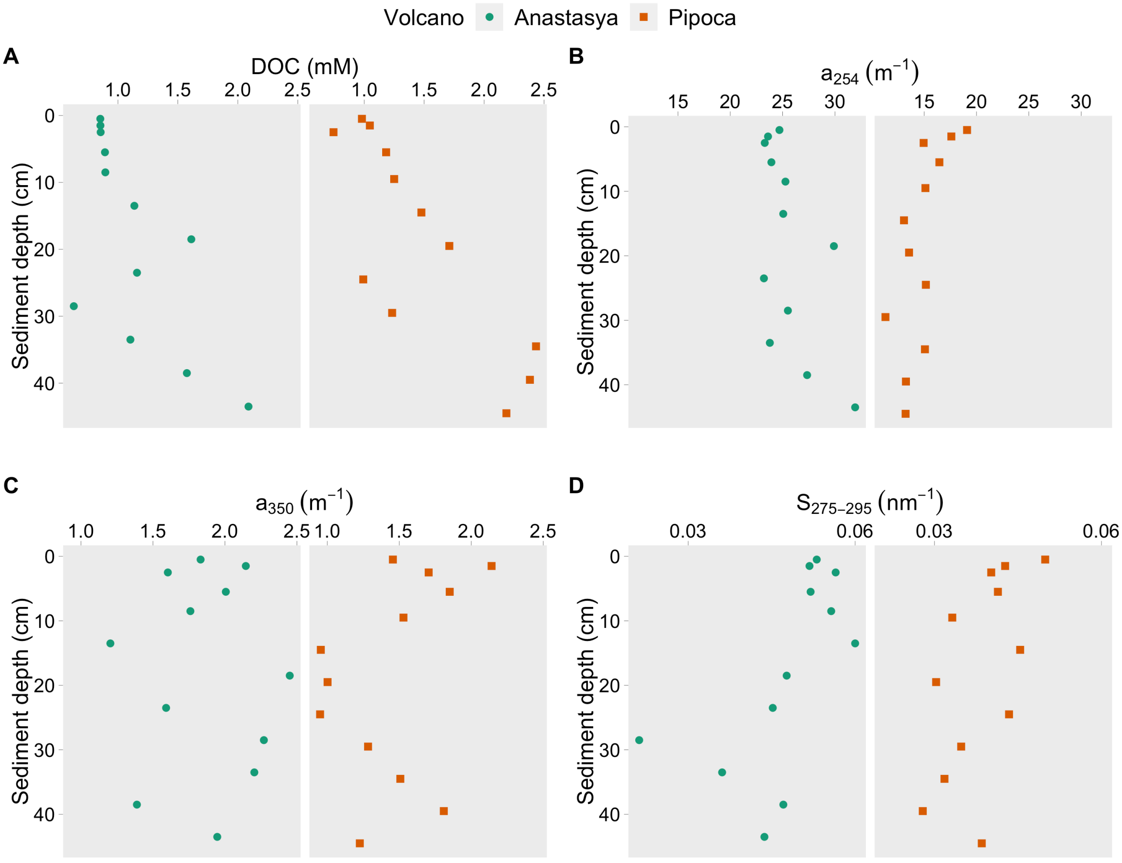

Fig. S1. Pore water profiles of dissolved organic carbon (DOC), absorption coefficients (a_{254} and a_{350}) and the spectral slope ($S_{275-295}$) in Anastasya and Pipoca during December 2016.

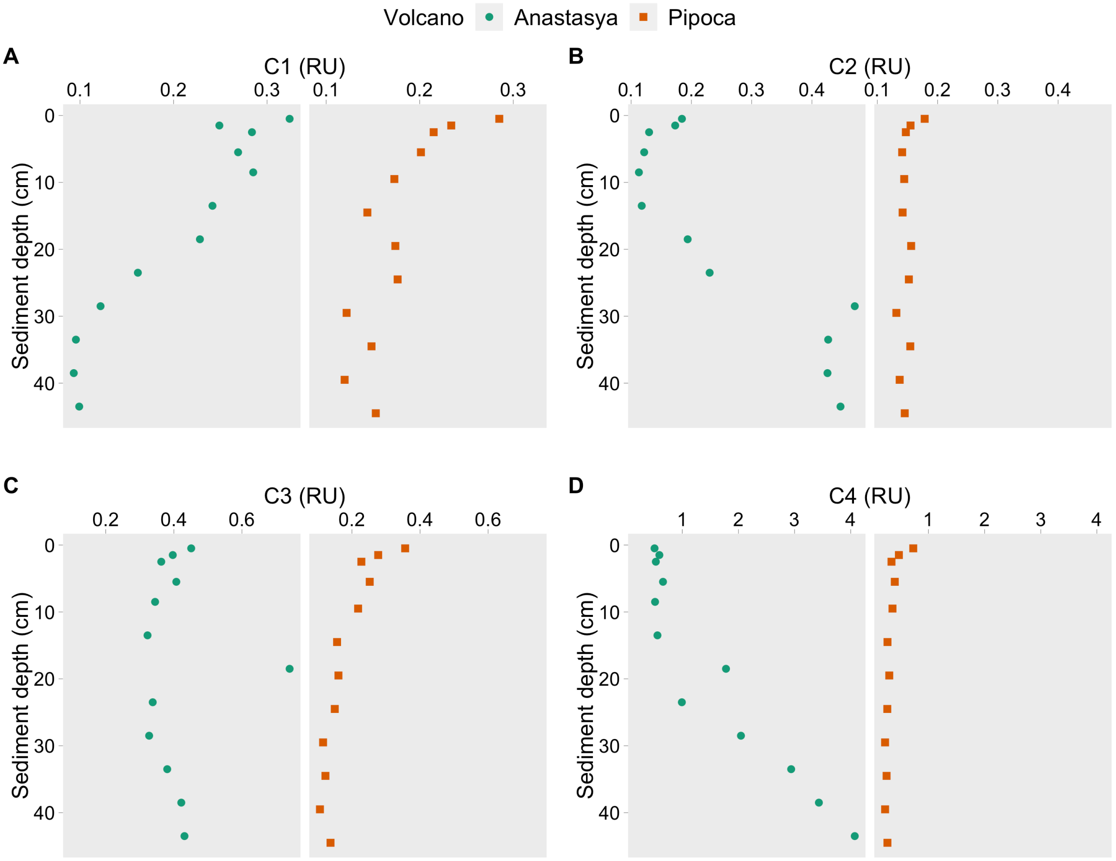

Fig. S2. Pore water profiles of the four fluorescent components (C1 to C4) in Anastasya and Pipoca during December 2016.

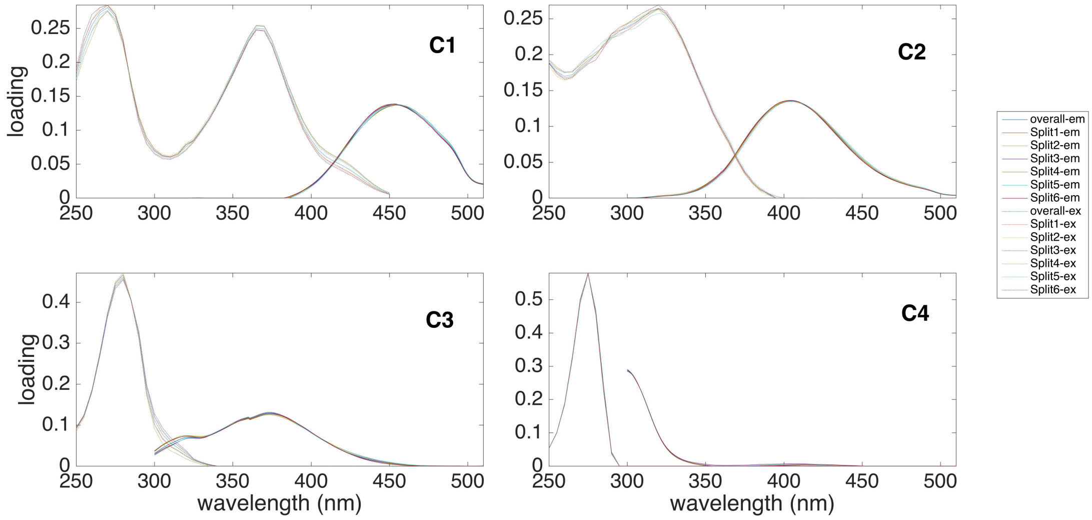

Fig. S3. Split-half validation of the four PARAFAC components validated in the pore water model.

June 2016

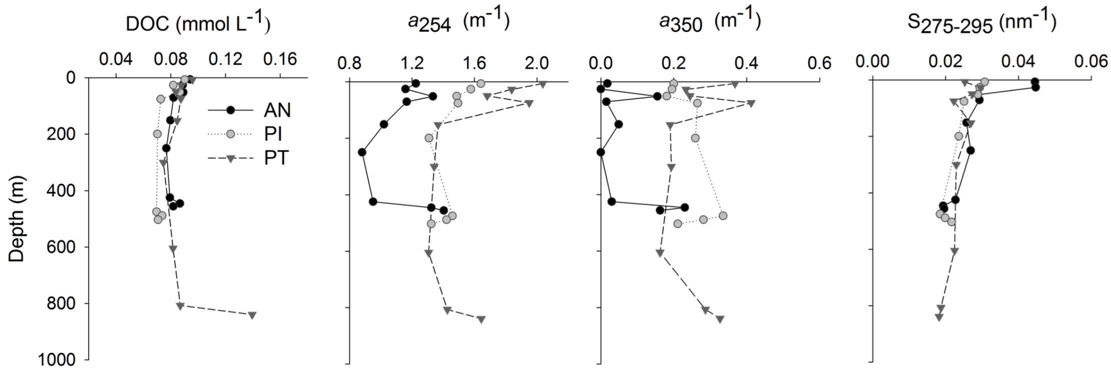

December 2016

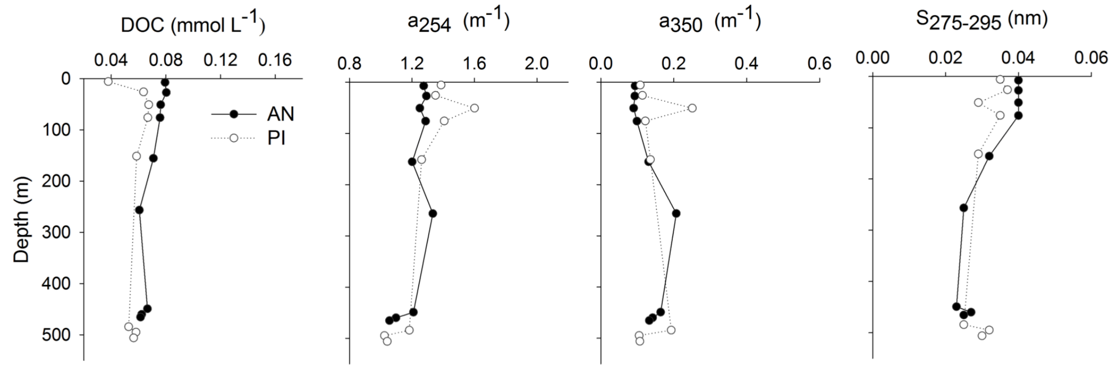

Fig. S4. Seawater vertical profiles of dissolved organic carbon (DOC), absorption coefficients (a_{254} and a_{350}) and the spectral slope ($S_{275-295}$) above AN: Anastasya, PI: Pipoca and PT: St. Petersburg during June and December 2016.

June 2016

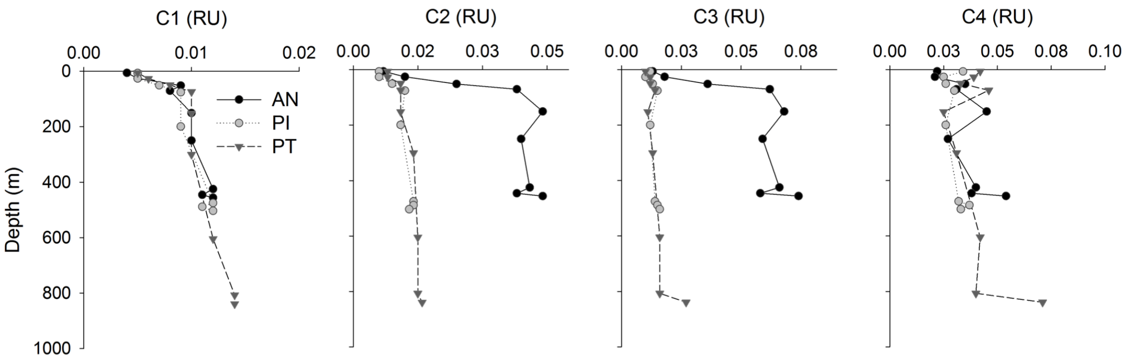

December 2016

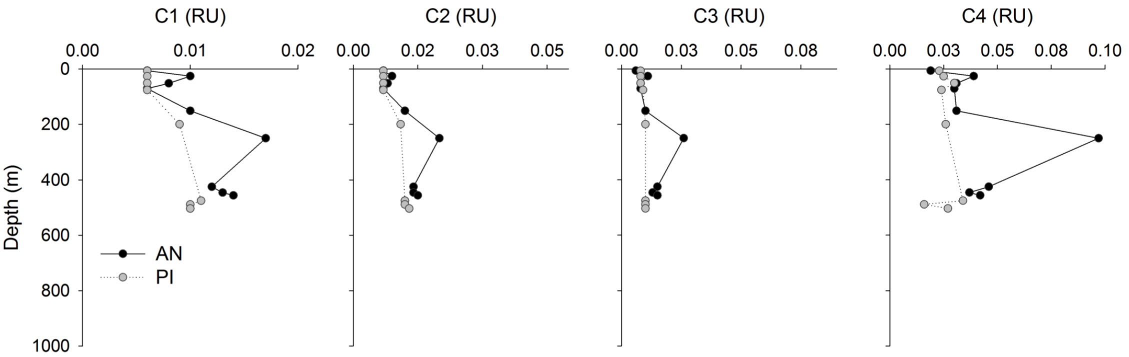

Fig. S5. Seawater vertical profiles of fluorescent components (C1 to C4) above AN: Anastasya, PI: Pipoca and PT: St. Petersburg during June and December 2016.

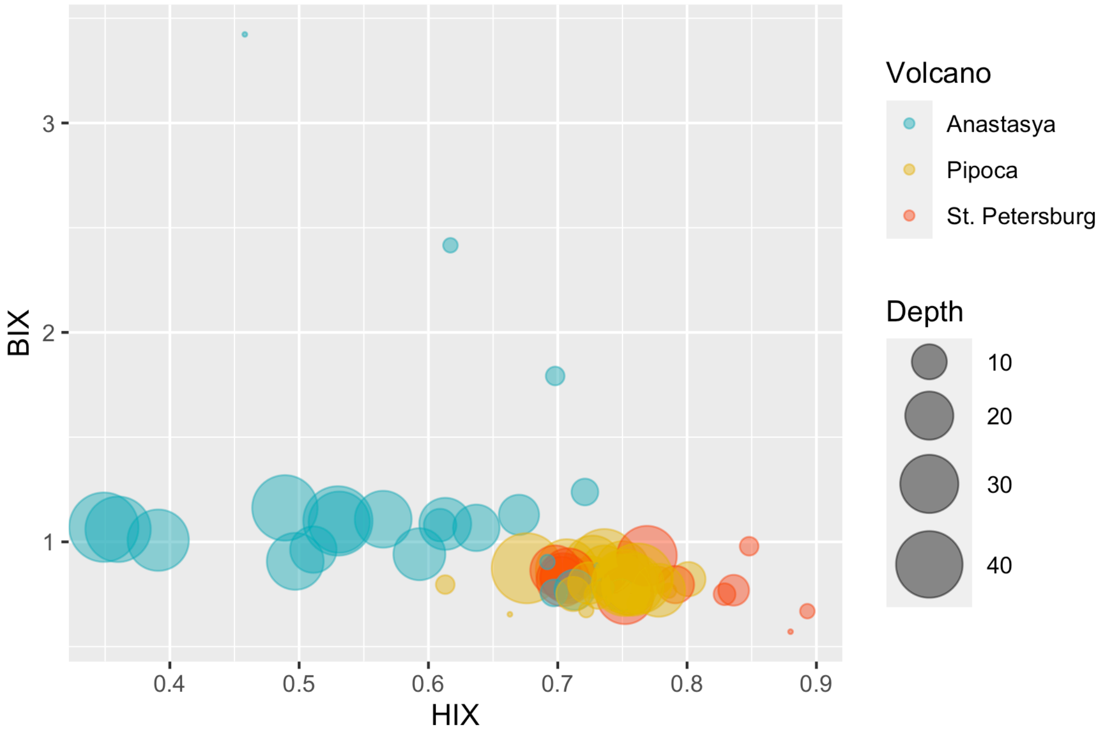

Fig. S6. Biplot of HIX versus BIX for each mud volcano during the study period. Size of circles represents depth.

Table S1. Location and morphological parameters of the three mud volcanoes in the study area. Data from Palomino et al.[1] 2016 and Sánchez-Guillamón,[2] 2019.

Name	Coordinates	Max/min depth (m)	Height (m)	Area (km^2)	Cone shape
Anastasya	7.15 ºW, 36.52 ºN	555/457	98	3.26	symmetric
Pipoca	7.20 ºW, 36.46 ºN	762/503	115	4.54	asymmetrical
San Petersburg	7.03 ºW, 35.89 ºN	935/1000	110	2.98	asymmetrical

Table S2. Excitation (Ex.) and Emission (Em.) maxima of the four PARAFAC components in the pore water model (n = 58). Matching with Openfluor database with a TCC > 0.97 are shown (* TCC = 0.95).

	Ex/Em	Openfluor matching	Description	Zone
C1	270	C3, Chen et al.[3]	Terrestrial, humic	Arctic sediments
	(365)/454	C3, Cawley et al.[4]	Humic-like	Shark Bay
		C3, Graeber et al.[5]	Humic-like	Agricultural
		C3, Osburn & Stedmon[6]	Terrestrial, humic	Baltic Sea
		C3, Murphy et al.[7]	-	-
		C3, Amaral et al. [8]	Terrestrial, humic	Gulf of Cádiz
C2	320/405	C1, Amaral et al.[8]	Ubiquitous humic-like	Gulf of Cádiz
		C2, Schittich et al. [9]	Humic-like	Groundwater
		C2, Catalá et al.[10]	Marine	Global ocean
		C3, Kowalczuk et al. [11]	Marine/microbial	Atlantic Bight
		C6, Stedmon et al.[12]	Marine	Antarctic sea ice
		C2, Kothawala et al.[13]	Marine	Lakes, incubations
		C2 Kulkarni et al.[14]	Terrestrial	Groundwater
		C1, Wünsch et al.[15]	-	Arctic Fjords
		C2, Asmala et al.[16]	-	Coastal zone
		C2, Hambly et al.[17]	Ubiquitous	Aquaculture
		C2, Chen et al.[18]	Marine	Arctic waters
		C2, Chen et al.[3]	Microbial/Marine	Arctic pore water
		C2, Osburn et al.[19]	Microbial humic-like	Estuarine and Coastal
		C6, Yamashita et al.[20]	Ubiquitous humic-like	Subtropical wetlands
		C2, Dalmagro et al.[21]	Ubiquitous humic-like	Streams riparian forest

		C4, Podgorski et al.[22]	Microbial/Marine	Groundwater oil derived
C3	280/375	C3, Sharma et al.[23]	Complex nature	Soil organic matter
		C5*, Amaral et al.[8]	PAH-protein like	Gulf of Cádiz
C4	275/303	C1, Murphy et al. [24]	Tyrosine	Ocean
		C4 Kowalczuk et al.[25]	Tyrosine	Coastal
		C5, Osburn et al.[19]	Tyrosine	Coastal
		C6*, Amaral et al.[8]	Tyrosine	Gulf of Cádiz

Table S3. Linear relationships between FDOM components and with DOM variables in the three MVs (AN: Anastasya, PI: Pipoca, n = 24 and PT: St. Petersburg, n = 12). The coefficient of determination R^2 is indicated when $p < 0.01$ and ns is $p > 0.01$. A negative R^2 is indicative of an inverse relationship between variables.

	AN	PI	PT
C1-C2	-0.71	0.71	0.60
C1-C3	ns	0.95	0.65
C1-C4	-0.53	0.82	0.62
C2-C4	ns	0.53	0.56
C2-C3	ns	0.52	0.50
C3-C4	ns	0.92	0.96
a_{254}-C1	0.31	0.81	0.35
a_{254}-C2	ns	0.38	0.36
a_{254}-C3	0.31	0.60	0.65
a_{254}-C4	ns	0.52	0.73
$S_{275-295}$-C1	0.56	ns	ns
$S_{275-295}$-C2	-0.75	ns	ns
$S_{275-295}$-C4	-0.53	ns	ns
HIX-C1	0.59	ns	ns
HIX-C2	-0.36	ns	ns
HIX-C4	-0.94	ns	ns
BIX-C1	ns	-0.57	ns
BIX-C2	0.41	-0.37	ns
BIX-C3	ns	-0.60	ns
BIX-C4	0.33	-0.58	ns

References

1. Palomino, D. *et al.* Multidisciplinary study of mud volcanoes and diapirs and their relationship to seepages and bottom currents in the Gulf of Cádiz continental slope (northeastern sector). *Mar. Geol.* **378**, 196–212 (2016).
2. Sánchez-Guillamón, O. Deep Submarine volcanoes in two geodynamic settings (Canary Basin and Gulf of Cádiz): Morphology and shallow structure. (2019).
3. Chen, M. *et al.* Production of fluorescent dissolved organic matter in Arctic Ocean sediments. *Sci. Rep.* **6**, 1–10 (2016).
4. Cawley, K. M., Ding, Y., Fourqurean, J. W. & Jaffé, R. Characterising the sources and fate of dissolved organic matter in Shark Bay , Australia : A preliminary study using a preliminary study using optical properties and stable carbon isotopes. **63**, 1098–1107 (2012).
5. Graeber, D., Gelbrecht, J., Pusch, M. T., Anlanger, C. & von Schiller, D. Agriculture has changed the amount and composition of dissolved organic matter in Central European headwater streams. *Sci. Total Environ.* **438**, 435–446 (2012).
6. Osburn, C. L. & Stedmon, C. A. Linking the chemical and optical properties of dissolved organic matter in the Baltic-North Sea transition zone to differentiate three allochthonous inputs. *Mar. Chem.* **126**, 281–294 (2011).
7. Murphy, K. R., Stedmon, C. A., Graeber, D. & Bro, R. Fluorescence spectroscopy and multi-way techniques. PARAFAC. *Anal. Methods* **5**, 6557–6566 (2013).
8. Amaral, V., Romera-Castillo, C. & Forja, J. Dissolved Organic Matter in the Gulf of Cádiz: Distribution and Drivers of Chromophoric and Fluorescent Properties. *Front. Mar. Sci.* **7**, 1–15 (2020).
9. Schittich, A. R. *et al.* Investigating Fluorescent Organic-Matter Composition as a Key Predictor for Arsenic Mobility in Groundwater Aquifers. *Environ. Sci. Technol.* **52**, 13027–13036 (2018).
10. Catalá, T. S. *et al.* Turnover time of fluorescent dissolved organic matter in the dark global ocean. *Nat. Commun.* **6**, (2015).
11. Kowalczuk, P. *et al.* Characterization of dissolved organic matter fluorescence in the South Atlantic Bight with use of PARAFAC model: Interannual variability. *Mar. Chem.* **113**, 182–196 (2009).
12. Stedmon, C. A., Thomas, D. N., Papadimitriou, S., Granskog, M. A. & Dieckmann, G. S. Using fluorescence to characterize dissolved organic matter in Antarctic sea ice brines. *J. Geophys. Res. Biogeosciences* **116**, 1–9 (2011).
13. Kothawala, D. N., von Wachenfeldt, E., Koehler, B. & Tranvik, L. J. Selective loss and preservation of lake water dissolved organic matter fluorescence during long-term dark incubations. *Sci. Total Environ.* **433**, 238–246 (2012).
14. Kulkarni, H. V., Mladenov, N., Johannesson, K. H. & Datta, S. Contrasting dissolved organic matter quality in groundwater in Holocene and Pleistocene aquifers and implications for influencing arsenic mobility. *Appl. Geochemistry* **77**, 194–205 (2017).
15. Wünsch, U. J. *et al.* Quantifying the impact of solid-phase extraction on chromophoric dissolved organic matter composition. *Mar. Chem.* **207**, 33–41 (2018).
16. Asmala, E. *et al.* Eutrophication Leads to Accumulation of Recalcitrant Autochthonous Organic Matter in Coastal Environment. *Global Biogeochem. Cycles* **32**, 1673–1687 (2018).
17. Hambly, A. C. *et al.* Characterising organic matter in recirculating aquaculture

systems with fluorescence EEM spectroscopy. *Water Res.* **83**, 112–120 (2015).
18. Chen, M., Jung, J., Lee, Y. K. & Hur, J. Surface accumulation of low molecular weight dissolved organic matter in surface waters and horizontal off-shelf spreading of nutrients and humic-like fluorescence in the Chukchi Sea of the Arctic Ocean. *Sci. Total Environ.* **639**, 624–632 (2018).
19. Osburn, C. L. *et al.* Optical proxies for terrestrial dissolved organic matter in estuaries and coastal waters. *Front. Mar. Sci.* **2**, (2016).
20. Yamashita, Y., Scinto, L. J., Maie, N. & Jaffé, R. Dissolved Organic Matter Characteristics Across a Subtropical Wetland's Landscape: Application of Optical Properties in the Assessment of Environmental Dynamics. *Ecosystems* **13**, 1006–1019 (2010).
21. Dalmagro, H. J. *et al.* Streams with riparian forest buffers versus impoundments differ in discharge and DOM characteristics for pasture catchments in Southern Amazonia. *Water (Switzerland)* **11**, 1–20 (2019).
22. Podgorski, D. C. *et al.* Examining Natural Attenuation and Acute Toxicity of Petroleum-Derived Dissolved Organic Matter with Optical Spectroscopy. *Environ. Sci. Technol.* **52**, 6157–6166 (2018).
23. Sharma, P. *et al.* Green manure as part of organic management cycle: Effects on changes in organic matter characteristics across the soil profile. *Geoderma* **305**, 197–207 (2017).
24. Murphy, K. R., Ruiz, G. M., Dunsmuir, W. T. M. & Waite, T. D. Optimized parameters for fluorescence-based verification of ballast water exchange by ships. *Environ. Sci. Technol.* **40**, 2357–2362 (2006).
25. Kowalczuk, P., Tilstone, G. H., Zabłocka, M., Röttgers, R. & Thomas, R. Composition of dissolved organic matter along an Atlantic Meridional Transect from fluorescence spectroscopy and Parallel Factor Analysis. *Mar. Chem.* **157**, 170–184 (2013).
26. Sierra, A. *et al.* Methane dynamics in the coastal – Continental shelf transition zone of the Gulf of Cadiz. *Estuar. Coast. Shelf Sci.* **236**, (2020).
